# GastroBot: a Chinese gastrointestinal disease chatbot based on the retrieval-augmented generation

**DOI:** 10.3389/fmed.2024.1392555

**Published:** 2024-05-22

**Authors:** Qingqing Zhou, Can Liu, Yuchen Duan, Kaijie Sun, Yu Li, Hongxing Kan, Zongyun Gu, Jianhua Shu, Jili Hu

**Affiliations:** ^1^School of Medical Information Engineering, Anhui University of Chinese Medicine, Hefei, China; ^2^Center for Xin’an Medicine and Modernization of Traditional Chinese Medicine of IHM, Anhui University of Chinese Medicine, Hefei, China

**Keywords:** retrieval-augmented generation, gastrointestinal disease, natural language process, large language models, embedding model, chatbot, question-answering, GPT

## Abstract

**Introduction:**

Large Language Models (LLMs) play a crucial role in clinical information processing, showcasing robust generalization across diverse language tasks. However, existing LLMs, despite their significance, lack optimization for clinical applications, presenting challenges in terms of illusions and interpretability. The Retrieval-Augmented Generation (RAG) model addresses these issues by providing sources for answer generation, thereby reducing errors. This study explores the application of RAG technology in clinical gastroenterology to enhance knowledge generation on gastrointestinal diseases.

**Methods:**

We fine-tuned the embedding model using a corpus consisting of 25 guidelines on gastrointestinal diseases. The fine-tuned model exhibited an 18% improvement in hit rate compared to its base model, gte-base-zh. Moreover, it outperformed OpenAI’s Embedding model by 20%. Employing the RAG framework with the llama-index, we developed a Chinese gastroenterology chatbot named “GastroBot,” which significantly improves answer accuracy and contextual relevance, minimizing errors and the risk of disseminating misleading information.

**Results:**

When evaluating GastroBot using the RAGAS framework, we observed a context recall rate of 95%. The faithfulness to the source, stands at 93.73%. The relevance of answers exhibits a strong correlation, reaching 92.28%. These findings highlight the effectiveness of GastroBot in providing accurate and contextually relevant information about gastrointestinal diseases. During manual assessment of GastroBot, in comparison with other models, our GastroBot model delivers a substantial amount of valuable knowledge while ensuring the completeness and consistency of the results.

**Discussion:**

Research findings suggest that incorporating the RAG method into clinical gastroenterology can enhance the accuracy and reliability of large language models. Serving as a practical implementation of this method, GastroBot has demonstrated significant enhancements in contextual comprehension and response quality. Continued exploration and refinement of the model are poised to drive forward clinical information processing and decision support in the gastroenterology field.

## 1 Introduction

In recent years, there has been significant advancement in large language models (LLMs), with notable models like ChatGPT demonstrating remarkable performance in question answering, summarization, and content generation (1–3). These models exhibit robust generalization not only within natural language processing (NLP) tasks but also across various interdisciplinary domains (4). However, models akin to ChatGPT, trained on general datasets, lack specific optimizations for clinical applications and encounter issues in question generation characterized by “perceptual illusions” and “unrealistic” features (5–7), potentially resulting in the provision of incomplete or inaccurate information and posing inherent risks (8, 9). To specialize LLMs, three methods have been proposed: optimizing the original LLM model (10), employing prompt engineering (11–13), and Retrieval-Augmented Generation (RAG) (14).

RAG, introduced in 2020, is a retrieval-augmented technique capable of fetching information from external knowledge sources, thus significantly enhancing answer accuracy and relevance (15). In recent years, RAG technology has proven effective in the biomedical field (16). Wang et al. (17) developed Almanac, which improved medical guideline retrieval. Ge et al. (18) created Li Versa for liver disease queries, while Ranjit et al. (19) applied RAG to radiology reports. Yu et al. (20) utilized RAG for diagnosing heart disease and sleep apnea, whereas Lozano et al. (21) and Manathunga et al. (22) applied it to medical literature and education.

This study focuses on the application of RAG in the field of clinical gastroenterology in China, aiming to address the issue associated with the continuous increase in the infection rate of *Helicobacter pylori* and the rising incidence of gastric cancer (23, 24). Given the substantial patient population with gastrointestinal diseases and the complexity of diagnosis and treatment, the “illusions” brought about by LLMs may pose additional challenges to the diagnosis and treatment of gastrointestinal diseases (5, 7). Integrating RAG is crucial for enhancing the accuracy of clinical practitioners in managing these diseases and can effectively mitigate this issue.

**Figure 1:**
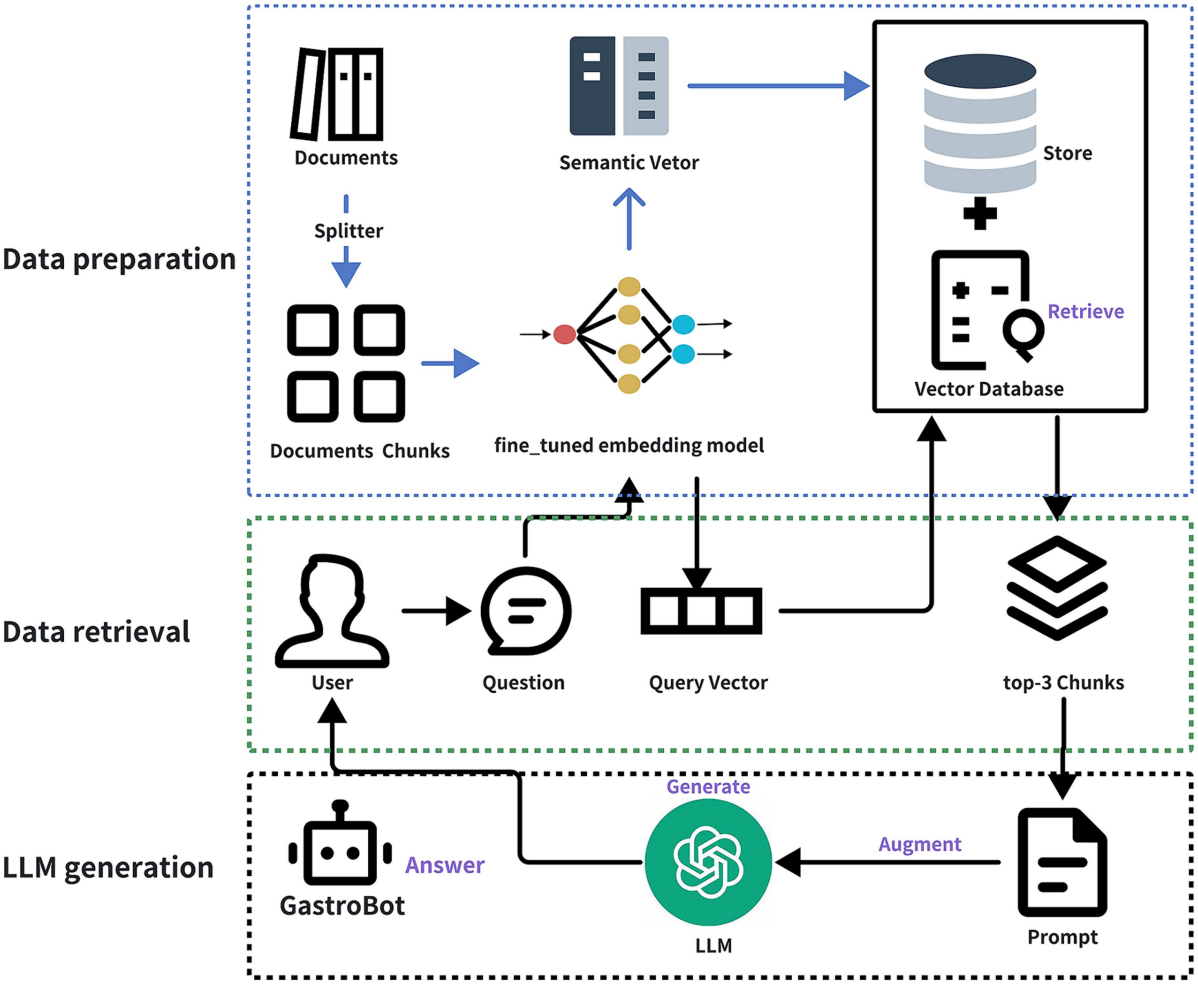
The overview of GastroBot. The process begins with the data preparation stage, where documents are initially split using a Splitter, dividing them into multiple document chunks. Subsequently, each chunk is encoded using the fine-tuned embedding model, producing semantic vectors stored in a Vector Database. Moving on to the data retrieval stage, the user inputs a question (User Question), and based on the question vector (Query Vector), the most relevant chunks (top-3 Chunks) are retrieved from the vector database. Finally, in the LLM generation phase, the large model generates answers by combining the top-3 chunks and the prompt, submitting them together to the LLM to obtain the final answer (Answer).

The aim of this study is to leverage RAG and large-scale language models, utilizing 25 guidelines on gastrointestinal diseases and 40 recent gastrointestinal literature sources as external knowledge bases, to develop a dedicated chatbot for gastrointestinal diseases named GastroBot. Furthermore, to enhance the relevance between retrieved content and user queries, this study conducted domain-specific fine-tuning of the embedding model tailored to gastrointestinal diseases, directly enhancing the performance of RAG. GastroBot is capable of providing precise diagnosis and treatment recommendations for gastrointestinal patients, thereby improving treatment efficacy. Figure 1 illustrates the comprehensive workflow of GastroBot.

In summary, the main contributions can be summarized as follows:

- We have created a specialized dataset named the “EGD Database” specifically for Chinese gastrointestinal diseases.
- We performed domain-specific fine-tuning of the embedding model to enhance retrieval performance for gastrointestinal diseases.
- We utilized 25 gastrointestinal disease guidelines and 40 related literature articles as the knowledge base to develop a gastrointestinal disease chatbot named GastroBot using RAG and LLM.

## 2 Materials and methods

### 2.1 Dataset and data preprocessing

In order to develop a gastrointestinal chatbot tailored for the Chinese context, we initially sourced 25 clinical guideline documents related to gastrointestinal diseases from the Chinese Medical Journal Full-text Database.^1^ These guidelines were selected based on their alignment with the most current official guidelines in the field, ensuring comprehensive coverage across various dimensions. Additionally, we integrated the latest literature on gastroenterology from the China National Knowledge Infrastructure (CNKI) database,^2^ categorized under the discipline of digestive system diseases. These articles covered a range of topics including gastroesophageal reflux disease, *Helicobacter pylori*, clinical observations, *H. pylori* infection, and peptic ulcer diseases, all published in 2024, totaling 40 articles.

Subsequently, we conducted data preprocessing on the collected dataset, removing elements such as English abstracts and references that were irrelevant to our research objectives. Given RAG’s inability to process images, all image data were excluded during this preprocessing stage. The resulting refined dataset was named the “EGD Database,” with EGD standing for Expert Guidelines for Gastrointestinal Diseases.

### 2.2 Experiment

The experimental section comprises two crucial steps aimed at developing a dedicated Chinese gastrointestinal disease chatbot, named GastroBot, for knowledge-based question answering on gastrointestinal diseases. The initial steps involve fine-tuning the embedding model specifically for gastrointestinal diseases. Subsequently, the LlamaIndex^3^ is employed to construct the RAG pipeline.

#### 2.2.1 Fine-tuning embedding model

The objective of fine-tuning is to strengthen the correlation between retrieved content and queries. Fine-tuning the embedding model aims to optimize the influence of retrieved content on generating outputs. Particularly in the medical domain, characterized by evolving or rare terminology, these tailored embedding techniques can enhance retrieval relevance. The GTE (25) embedding model is renowned for its high performance. In this study, the gte-base-zh model from Alibaba DAMO Academy served as the foundational embedding model and underwent domain-specific fine-tuning.

**Figure 2:**
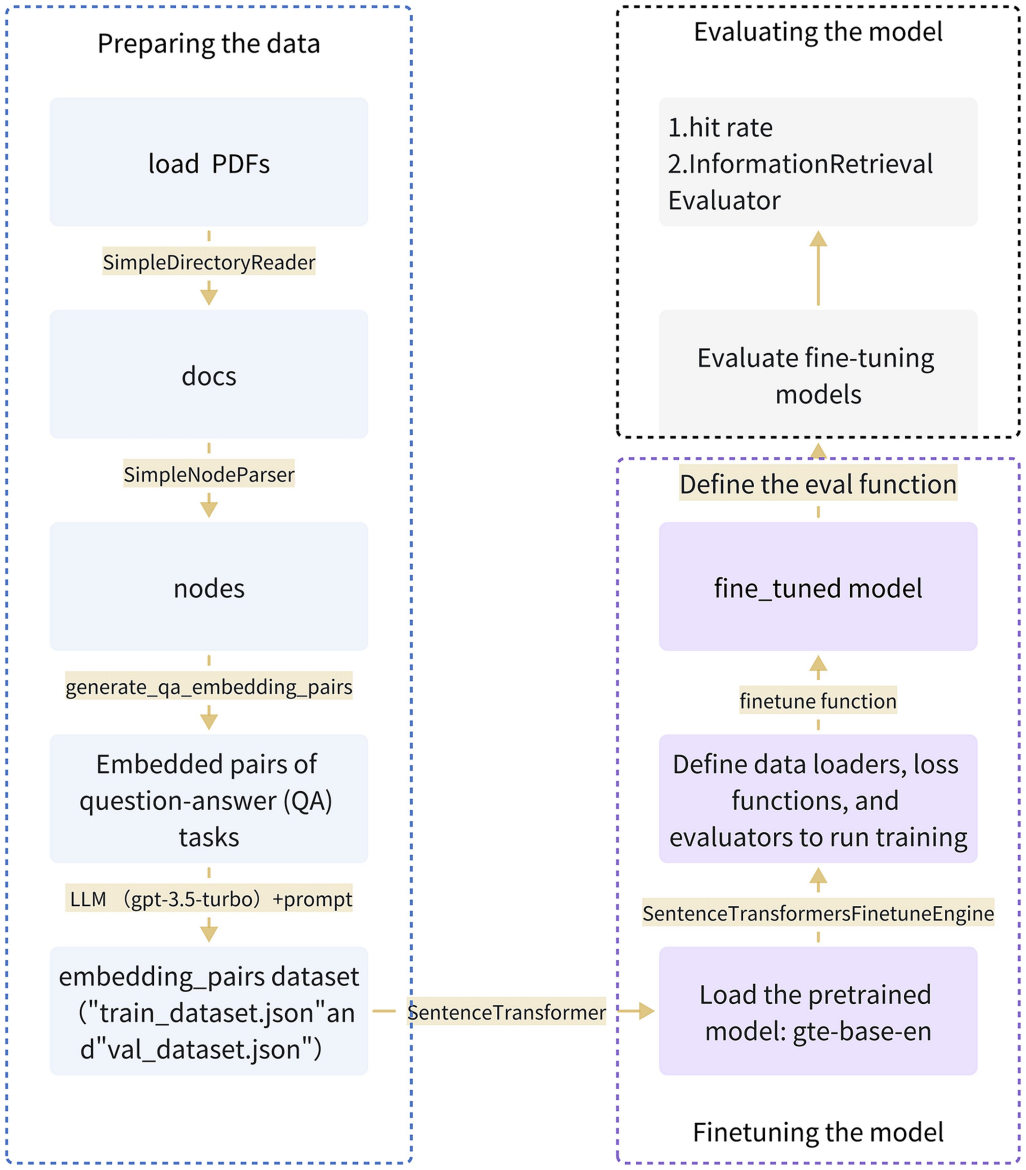
Provides a comprehensive overview of the entire process involved in fine-tuning the embedding model specific to the Chinese gastroenterology domain. The process encompasses three key steps: data preparation, model fine-tuning, and model evaluation.

For fine-tuning the gte-base-zh model, we employed GPT-3.5 Turbo to aid in generating question-answer pairs. During the fine-tuning process, the LLM generated questions based on document blocks, forming pairs with their respective answers. Then, the SentenceTransformersFinetuneEngine in LlamaIndex was utilized for fine-tuning. The fine-tuning of the customized Chinese gastrointestinal domain embedding model was accomplished through the steps illustrated in Figure 2.

##### 2.2.1.1 Data preprocessing in the fine-tuning stage

In this study, we leveraged the EGD database as the source of fine-tuning data and underwent a multi-step preprocessing procedure to adapt it into a corpus suitable for training and evaluation. Initially, we loaded cleaned and processed PDF files containing essential text information. Subsequently, the SimpleDirectoryReader tool was employed to extract data from designated files, generating a list containing all documents. Then, utilizing the SimpleNodeParser, we extracted meaningful node information from the documents. This parser was encapsulated within a callable function named load_corpus. After obtaining textual nodes, the data underwent transformation and processing through the generate_qa_embedding_pairs function in LlamaIndex to produce QA pairs suitable for fine-tuning embedding models. Furthermore, we utilized gpt-3.5-turbo to define context information and questions, establishing prompt generation templates. The resulting output is an embedded pair dataset, saved as “train_dataset,” which includes “train_dataset.json” and “val_dataset.json.” This process laid the groundwork for subsequent training and evaluation of the fine-tuned Chinese gastrointestinal embedding model.

##### 2.2.1.2 Training the fine-tuned model

We chose the gte-base-zh model from the GTE series models trained by Alibaba DAMO Academy as the embedding model for the fine-tuning phase. Throughout the fine-tuning process, the SentenceTransformersFinetuneEngine was employed to carry out various subtasks. This involved constructing a pre-training model using SentenceTransformer, defining a data loader responsible for loading the training dataset and parsing it into queries, corpus, and relevant_docs. Leveraging the gte-base-zh model, the engine mapped node_ids from relevant_docs to text nodes in the corpus and compiled a list of Input Examples. Training employed the multiple negatives ranking (MNR) loss from sentence_transformers, with an evaluator monitoring the model’s performance using the eval dataset throughout training. The entire process was seamlessly integrated into the training pipeline, encapsulated within the SentenceTransformersFinetuneEngine in LlamaIndex, and executed by invoking its fine-tuning function.

#### 2.2.2 Implementing retrieval-augmented generation with Llamaindex

The LlamaIndex framework is employed to construct the pipeline for RAG implementation. Initially, data loading is conducted to facilitate subsequent experiments. The entire document is segmented into smaller text units termed nodes, facilitating processing within the LlamaIndex framework. Using the SimpleNodeParser, the loaded documents are parsed and transformed into these nodes. Following this, within the global configuration object, the embedding model and LLM are explicitly specified. The chosen embedding model is fine-tuned for gastrointestinal diseases, streamlining the conversion of text into vector representations crucial for subsequent computations. The gpt-3.5 turbo is selected as the generative LLM and is utilized throughout the process for answer generation. Finally, three core components—index, retriever, and query engine—are instantiated via the LlamaIndex framework, collectively supporting question-answering functionality based on user data or documents. The index serves as a data structure for swiftly retrieving information pertinent to user queries from external documents. This is accomplished through the vector storage index, which generates vector embeddings for the text of each node. The retriever is responsible for acquiring and retrieving information relevant to user queries, while the query engine, built upon the index and retriever, furnishes a universal interface for posing questions to the data.

##### 2.2.2.1 Data preparation

###### 2.2.2.1.1 Load data

We are populating GastroBot’s knowledge base with a curated selection of 25 guidelines on gastrointestinal diseases and 40 research papers on gastroenterology. Leveraging Llamaindex’s SimpleDirectoryReader and PDFReader classes facilitates the loading and extraction of data from PDF documents, ensuring a streamlined dataset that prioritizes information pertinent to clinical gastroenterology.

###### 2.2.2.1.2 Chunking

Subsequently, in the text segmentation step, once the data extraction is complete, the document is partitioned into multiple text blocks referred to as chunks within LlamaIndex. Each chunk’s size is defined as 512 characters. Although the default ID for each node is a randomly generated text string, we have the flexibility to format it into a specific pattern as required.

###### 2.2.2.1.3 Embedding

Each chunk undergoes encoding using the fine-tuned embedding model to generate semantic vectors that encapsulate nuanced information within the captured segments. This fine-tuned embedding model excels particularly well in capturing specialized vocabulary associated with gastrointestinal diseases. Vectorization is pivotal as it transforms text data into a matrix of vectors, directly influencing the effectiveness of subsequent retrieval operations. While existing generic embedding models may serve adequately in many scenarios, in the medical domain, where rare specialized vocabulary or terminology is prevalent, we opted to fine-tune GTE to suit our specific application needs and enhance retrieval efficiency.

###### 2.2.2.1.4 Vector database

The semantic vectors generated are stored within the Vector Database, establishing an indexed repository optimized for swift and semantically aligned searches. This Vector Database forms the cornerstone for efficient retrieval in the subsequent phases of the RAG model. The intricacies of these steps are elaborated upon in the data preparation section of Figure 1.

##### 2.2.2.2 Building RAG

Having completed the data preparation phase, the second step involves the selection of the embedding model and LLM. The embedding model is tasked with generating vector embeddings for each text chunk, for which we employ a fine-tuned embedding model. Meanwhile, the LLM handles user queries and related text chunks, producing contextually relevant answers. To achieve this, we opt to utilize the gpt-3.5-turbo model via API calls. Both models collaborate synergistically within the service framework, playing indispensable roles in the indexing and querying processes. Subsequently, in the third step, we call upon LlamaIndex to construct the index, retriever, and query engine—these three pivotal components collectively facilitate question-answering based on user data or documents.

The index facilitates swift retrieval of information relevant to user queries directly from the external knowledge base. This is achieved through the creation of vector embeddings for the text of each node within the vector storage index. The retriever’s role is to acquire and retrieve information pertinent to user queries, while the query engine, positioned atop the index and retriever, offers a universal interface for posing inquiries to the data. The fundamental implementation of RAG, based on LlamaIndex, streamlines this process.

When a user poses a question, it undergoes conversion into a vector representation. Using this query vector, the most relevant segments (top-3 chunks) are retrieved from the vector database, constituting the Data retrieval phase depicted in Figure 1. Following this, the top-3 chunks, along with the prompt, are fed into the gpt-3.5-turbo model for answer generation, culminating in the final answer, as illustrated in the LLM generation phase of Figure 1. Throughout the process delineated in Figure 1, the user query is embedded into the same vector space as the additional context retrieved from the vector database, facilitating similarity-based search and returning the most proximate data objects, denoted as retrieval (labeled as Retrieve in the figure). The amalgamation of the user query and the supplementary context obtained from the prompt template is referred to as augmentation (labeled as Augment in the figure). Ultimately, the augmented prompt is input into the LLM for answer generation, a step termed generation (labeled as Generate in the figure).

#### 2.2.3 Comparative experiment

To demonstrate the superior performance of GastroBot, we conducted a comparative analysis between GastroBot and three baseline models utilizing RAG. When selecting these comparative models, we considered their scale, performance metrics, and diversity. Firstly, we selected Llama2 (26), an open-source language model that consistently outperforms other models across various external benchmark tests, including inference, encoding, proficiency, and knowledge evaluation. Secondly, we included ChatGLM-6B (27) and Qwen-7B (28), representing the latest advancements in Chinese artificial intelligence, as additional comparative models for this study, both demonstrating robust capabilities across multiple natural language processing tasks. We randomly selected 20 questions related to gastrointestinal diseases and compared the answers generated by GastroBot with those generated by the other three models. The performance of GastroBot relative to the other three models will be evaluated using human assessment methods.

### 2.3 Evaluation

#### 2.3.1 Embedding model evaluation

In this section, we will evaluate three different embedding models: OpenAI text-embedding-ada-002 (29), gte-base-zh, and our fine-tuned embedding model. We will employ two distinct evaluation methods:

Hit rate (30): Conducting a straightforward top-k retrieval for each query/relevant_doc pair. A retrieval is considered successful if the search result includes the relevant_doc, defining it as a “hit.” The hit ratio is shown in Equation (1):

(1) $$HR=\frac{1}{S}\sum_{i=1}^{S}hit\left(i\right)$$

S denotes the total number of query/relevant document pairs, representing the count of user demands. The function $hit\left(i\right)$ serves as an indicator; its value is 1 if the relevant document for the i−th query is in the top-k search results, and 0 otherwise.

Information Retrieval Evaluator: A comprehensive metric suite provided by the LlamaIdex for the evaluation of open-source embeddings. This class evaluates an Information Retrieval (IR) (31) setting. Given a set of queries and a large corpus set. It will retrieve for each query the top-k most similar document. It measures Mean Reciprocal Rank (MRR) (32), Recall@k, and Normalized Discounted Cumulative Gain (NDCG) (33, 34).

#### 2.3.2 Using RAGAs to evaluate RAG

Ragas (35) is a large-scale model evaluation framework designed to assess the effectiveness of Retrieval-Augmented Generation (RAG). It aids in analyzing the output of models, providing insights into their performance on a given task.

To assess the RAG system, Ragas requires the following information:

Questions: Queries provided by users.

Answers: Responses generated by the RAG system (elicited from a large language model, LLM).

Contexts: Documents relevant to the queries retrieved from external knowledge sources.

Ground Truths: Authentic answers provided by humans, serving as the correct references based on the questions. This is the sole required input.

Once Ragas obtains this information, it will utilize LLMs to evaluate the RAG system.

Ragas’s evaluation metrics comprise Faithfulness, Answer Relevance, Context Precision, Context Relevancy, Context Recall, Answer Semantic Similarity, Answer Correctness, and Aspect Critique. For this study, our chosen evaluation metrics are Faithfulness, Answer Relevance, and Context Recall.

##### 2.3.2.1 Faithfulness

Faithfulness is evaluated by assessing the consistency of generated answers with the provided context, derived from both the answer itself and the context of retrieval. Scores are scaled from 0 to 1, with higher scores indicating greater faithfulness.

An answer is deemed reliable if all assertions within it can be inferred from the given context. To compute this value; a set of assertions needs identification from the generated answer, followed by cross-checking each assertion against the provided context. The Equation (2) for computing faithfulness is as follows:

(2) $$\begin{array}{c} F=\frac{\left|V\right|}{\left|S\right|} \end{array}$$

Where |V| represents the number of statements supported by LLM, and |S| denotes the total number of statements.

##### 2.3.2.2 Answer relevance

To evaluate the relevance of answers: utilize LLM to generate potential questions and compute their similarity to the original question. The relevance score of an answer is determined by averaging the similarity between all generated questions and the original question.

Let the original question be q, and the answer to the question be $a_{s}\left(q\right)$ . The context segment relevant to question q is denoted *as* $\;\;c\left(q\right).$ If the claims presented in the answer can be inferred from the context, we assert that the answer $a_{s}\left(q\right)$ is faithful to the context $c\left(q\right)$ . To gauge credibility, we initially employ LLM to extract a set of statements $S\left(a_{s}\left(q\right)\right)$ . If the answer $a_{s}\left(q\right)$ directly and appropriately addresses the question, we consider it relevant. Particularly, our evaluation of answer relevance does not account for factual accuracy but penalizes incomplete or redundant information in the answers. To estimate answer relevance, given an answer $a_{s}\left(q\right)$ , we prompt LLM to generate n potential questions $q_{i}$ based on $a_{s}\left(q\right)$ . Subsequently, we use the text-embedding-ada-002 model from the OpenAI API to obtain embeddings for all questions. For each $q_{i}$ , we calculate the similarity $sim\left(q,q_{i}\right)$ with the original question q . The specific formula for Answer relevance is Equation (3):

(3) $$\begin{array}{c} AR=\frac{1}{n}\overset{n}{\underset{i=1}{\sum }}sim\left(q,q_{i}\right) \end{array}$$

This metric assesses the alignment between the generated answers and the initial question or instruction.

##### 2.3.2.3 Context recall

Context recall assesses how well the retrieved context aligns with the authentic answers provided by humans. It is calculated by comparing the ground truth with the retrieved context, with scores ranging from 0 to 1, where higher scores indicate better performance.

To estimate context recall based on the authentic answers, each sentence in the authentic answers is examined to determine its relevance to the retrieved context. Ideally, all sentences in the authentic answers should be relevant to the retrieved context. The context relevance score is calculated using the following Equation (4):

(4) $$\begin{array}{c} CR=\frac{\left|Groundtruthsentencesthat\;can\;be\;attributedtocontext\right|}{\left|Numberofsentencesingroundtruth\right|} \end{array}$$

This formula quantifies the proportion of sentences in the authentic answers that can be attributed to the retrieved context, providing a measure of how well the retrieved context aligns with the ground truth.

#### 2.3.3 Human evaluation

Although RAGAS has contributed to assessing the performance of RAG to some extent, human evaluation remains essential from the perspectives of safety, validation of professional knowledge, flexibility and adaptability, as well as ethical considerations. Therefore, this study incorporated human assessment. Our investigation presents the SUS (Safety, Usability, and Smoothness) evaluation method for human assessors SUS encompasses three dimensions: safety, usability, and smoothness (36). The “safety” dimension evaluates whether the model-generated content could potentially mislead users, posing health risks. Assessments from the “usability” dimension reflect the depth of professional expertise, while the “smoothness” dimension gauges the proficiency of the generated model functioning as an LLM. The skill enhancement program employs a three-tier scoring system, ranging from 1 (unsatisfactory) to 3 (good), with 2 indicating acceptable performance.

## 3 Results

### 3.1 Illustrative Q&A examples from GastroBot

**Figure 3:**
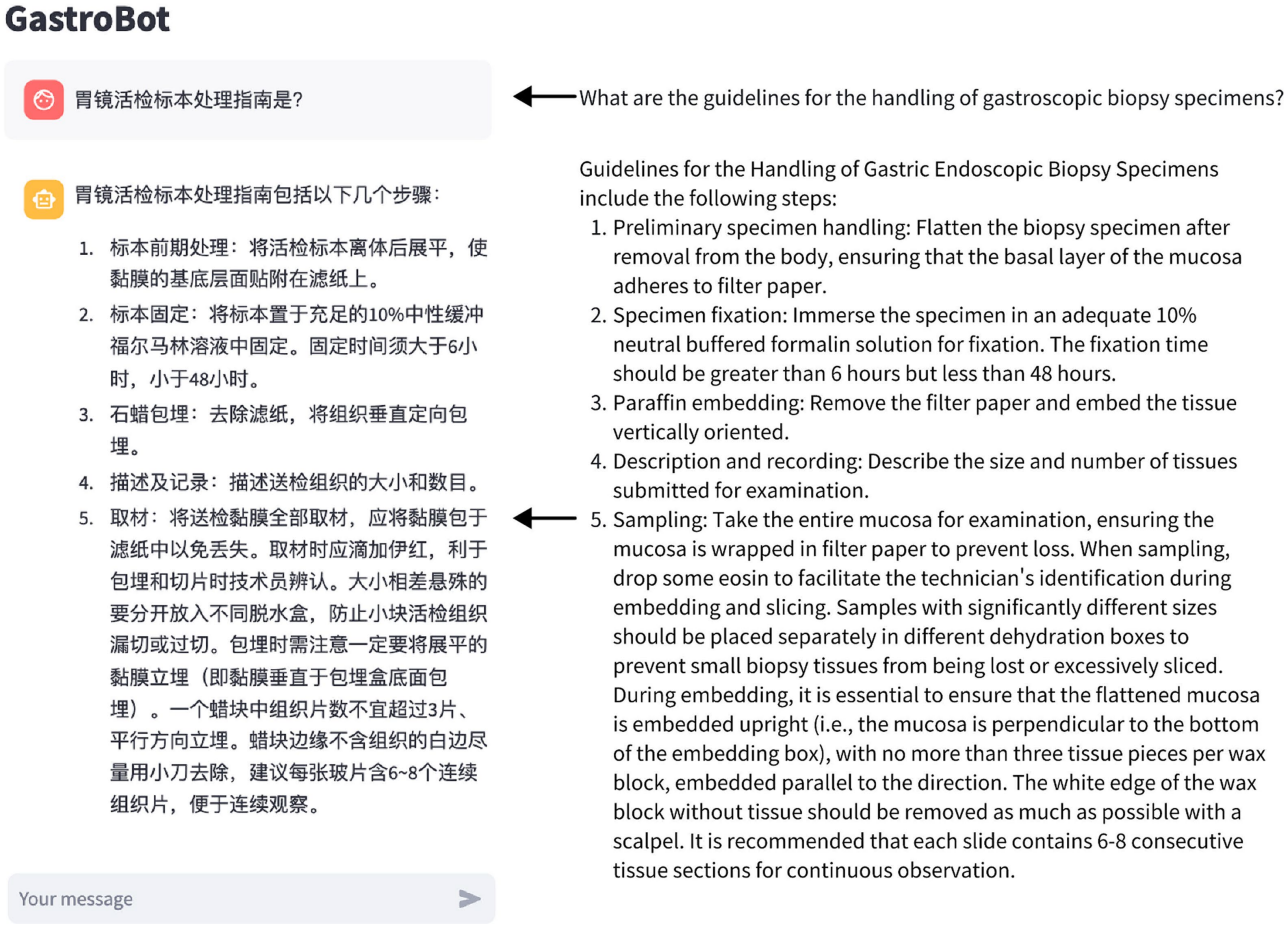
Depicts the chat interface of GastroBot, built using the Streamlit platform.

To demonstrate the capabilities of GastroBot, we developed a web application using Streamlit. This application enables users to input questions related to gastrointestinal diseases. Additionally, a question dialogue box is located below the main interface, allowing users to input their queries. The interface of the web application is depicted in Figure 3.

We randomly present five questions related to gastric diseases and generate answers using both GastroBot and ChatGPT. Table 1 compares the answers generated by GastroBot and ChatGPT for the five sample questions. Through this comparison, we observe that GastroBot’s answers are more precise, contextually relevant, and effectively mitigate the production of misleading information. For instance, in question 2, where the user in quires about the guidelines for handling gastric biopsy specimens, GastroBot provides a detailed response comprising five steps, each accompanied by precise explanations and requirements, and devoid of errors. In contrast, ChatGPT’s response offers only a series of steps without clear time references and lacks sufficient clarity in explanations.

**Table 1:** Five example questions and answers generated by GastroBot and ChatGPT.

问f	Queation	GastroBot生成的答案	Answers generated by GastroBot	ChatGPT生成的答案	Answers generated by ChatGPT
1.普通大众如何进行胃癌风险评估及筛查?	1. How can gastric cancer risk assessment and screening be performed in the general public?	普通大众可以通过胃癌风险评估和筛查来进行胃癌的预防。胃癌的筛查可以通过生物标志物初步筛查和内镜下精查的方式进行，逐级进行胃癌风险评估和筛查。常见的筛查手段包括血清生物标志物检查和内镜筛查等。血清生物标志物检查包括胃蛋白酶原、胃泌素-17及胃癌相关抗原等。而内镜筛查是一种常见的胃癌筛查手段。根据研究结果显示，内镜筛查组与不筛查组相比，胃癌死亡率下降了42%。因此，普通大众可以通过这些筛查手段来进行胃癌风险评估并进行预防。	Gastric cancer risk assessment and screening can be used to prevent gastric cancer in the general public. The screening of gastric cancer can be carried out by preliminary screening of biomarkers and precise examination under endoscopy, and gastric cancer risk assessment and screening can be carried out step by step. Common screening methods include serum biomarkers and endoscopic screening. Serum biomarkers included pepsinogen, gastrin-17 and gastric cancer associated antigens. Endoscopic screening is a common method for gastric cancer screening. According to the results of the study, there was a 42% reduction in gastric cancer mortality with endoscopic screening compared with no screening. Therefore, the general public can use these screening methods for gastric cancer risk assessment and prevention.	普通大众可通过关注家族史、定期体检和注意症状来进行胃癌风险评估。若家族中有胃癌病例，风险可能增加。定期体检包括体格检查和血液检查，可早期发现异常。注意症状如胃痛、体重下降需引起警惕。饮食保持均衡、戒烟戒酒也有助于降低风险。在有疑虑时，及早咨询医生，根据个人情况制定合适的筛查计划，如胃镜检查。	The general public can assess the risk of gastric cancer by paying attention to family history, regular physical examination, and paying attention to symptoms. The risk may increase if there are cases of gastric cancer in the family. Regular physical examinations, including physical examination and blood tests, can detect abnormalities early. Pay attention to symptoms such as stomach pain and weight loss. Eating a balanced diet and quitting smoking and alcohol can also help reduce the risk. When in doubt, consult a physician early to develop an appropriate screening program, such as gastroscopy, based on individual circumstances.
2.胃镜活检标本处理指南是?	2. What are the guidelines for the handling of gastroscopic biopsy specimens?	胃镜活检标本处理指南包括以下几个步骤:1.标本前期处理:将活检标本离体后展平，使黏膜的基底层面贴附在滤纸上。2、标本固定:将标本置于充足的10%中性缓冲福尔马林溶液中固定。固定时间须大于6小时，小于48小时。3.石蜡包埋:去除滤纸,将组织垂直定向包埋。4.描述及记录:描述送检组织的大小和数目。5.取材:将送检黏膜全部取材，应将黏膜包于滤纸中以免丢失。取材时应滴加伊红，利于包埋和切片时技术员辨认。大小相差悬殊的要分开放入不同脱水盒，防止小块活检组织漏切或过切。包埋时需注意一定要将展平的黏膜立埋(即黏膜垂直于包埋盒底面包埋)，一个蜡块中组织片数不宜超过3片、平行方向立埋。蜡块边缘不含组织的白边尽量用小刀去除，建议每张玻片含6-8个连续组织片，便于连续观察。	Guidelines for the Handling of Gastric Endoscopic Biopsy Specimens include the following steps:Preliminary specimen handling: Flatten the biopsy specimen after removal from the body, ensuring that the basal layer of the mucosa adheres to filter paper.Specimen fixation: Immerse the specimen in an adequate 10% neutral buffered formalin solution for fixation. The fixation time should be greater than 6 h but less than 48 h.Paraffin embedding: Remove the filter paper and embed the tissue vertically oriented.Description and recording: Describe the size and number of tissues submitted for examination.Sampling: Take the entire mucosa for examination, ensuring the mucosa is wrapped in filter paper to prevent loss. When sampling, drop some eosin to facilitate the technician’s identification during embedding and slicing. Samples with significantly different sizes should be placed separately in different dehydration boxes to prevent small biopsy tissues from being lost or excessively sliced. During embedding, it is essential to ensure that the flattened mucosa is embedded upright (i.e., the mucosa is perpendicular to the bottom of the embedding box), with no more than three tissue pieces per wax block, embedded parallel to the direction. The white edge of the wax block without tissue should be removed as much as possible with a scalpel. It is recommended that each slide contains 6–8 consecutive tissue sections for continuous observation.	胃镜活检标本处理包括标本采集、迅速送至实验室并湿润保存，使用固定剂如10%福尔马林固定组织，然后进行包埋在蜡块中。蜡块中的组织被切成薄切片，通常为3-5微米，随后进行染色，最常用的是H&E染色。染色后的切片放在显微镜下由专业医生或病理学家检查。医生根据显微镜检查的结果撰写病理报告，提供诊断或评估病变的信息。这一过程确保了标本的准确性和质量，对于胃癌等疾病的确诊和治疗规划至关重要。	Processing of gastroscopic biopsy specimens involves collection, rapid transport to the laboratory for moist storage, fixation with a fixative such as 10% formalin, and embedding in wax blocks. Tissue in wax blocks was cut into thin sections, usually 3–5 microns in size, and subsequently stained, most commonly with H&E. The stained sections were placed under a microscope and examined by a professional physician or pathologist. Physicians write pathology reports based on the findings of microscopic examination to provide information to diagnose or evaluate lesions. This process ensures the accuracy and quality of the specimen, which is essential for the diagnosis and treatment planning of diseases such as gastric cancer.
3.早期胃癌内镜治疗禁忌证是?	3. What are the contraindications of endoscopic therapy for early gastric cancer?	早期胃癌内镜治疗禁忌证包括明确淋巴结转移的早期胃癌、癌症侵犯固有肌层以及患者存在凝血功能障碍。此外，ESD的相对手术禁忌证还包括抬举征阴性，即指在病灶基底部的黏膜下层注射盐水后局部不能形成隆起，提示病灶基底部的黏膜下层与肌层之间已有粘连。在这种情况下进行ESD治疗，发生穿孔的危险性较高。然而，随着ESD操作技术的熟练，即使抬举征阴性也可以安全地进行ESD。	The contraindications of endoscopic treatment for EGC include lymph node metastasis, invasion of the muscularis propria, and coagulopathy. In addition, the relative surgical contraindications of ESD include negative lifting sign, which means that the local uplift cannot be formed after saline injection into the submucosa at the base of the lesion, suggesting that there is adhesion between the submucosa and the muscular layer at the base of the lesion. The risk of perforation is high when ESD is performed under these conditions. However, with proficiency in ESD manipulation techniques, ESD can be performed safely even with a negative lifting sign.	早期胃癌内镜治疗的禁忌证包括: 深度浸润: 如果癌症已经深度浸润，不适合内镜治疗。 淋巴结转移: 若有淋巴结转移，内镜治疗可能无法完全清除癌细胞。 广泛浸润: 如果癌变范围过大，内镜治疗难以覆盖所有异常区域。 合并其他疾病: 合并其他疾病，如出血倾向、严重心肺疾病，可能增加内镜治疗的风险。 患者不适合手术: 患者健康状况不适合手术的情况下，内镜治疗可能不合适。 在确定内镜治疗方案前，需仔细评估患者病情和整体健康状况，确保选择最适合的治疗方式。	Contraindications to endoscopic treatment of early gastric cancer include:Deep invasion: If the cancer is already deeply invasive, endoscopic treatment is not appropriate.Lymph node metastasis: If lymph node metastasis is present, endoscopic treatment may not completely remove cancer cells.Extensive invasion: If the area of cancer is too large, it is difficult to cover all abnormal areas with endoscopic treatment.Combined with other diseases: Combined with other diseases, such as bleeding tendency and severe cardiopulmonary diseases, may increase the risk of endoscopic treatment.Patient is not a Candidate for surgery: Endoscopic therapy may not be appropriate if the patient is medically unfit for surgery.Before deciding on endoscopic treatment, a careful assessment of the patient’s condition and overall health status is necessary to ensure that the most appropriate treatment is selected.
4.慢性胃炎的病因和分类是?	4. What are the causes and classifications of chronic gastritis?	慢性胃炎的病因包括H.pylori感染、自身免疫性胃炎、食物诱发的嗜酸细胞性胃肠炎、淋巴细胞性胃炎等。根据慢性胃炎的分类系统，可以将其分为慢性非萎缩性胃炎和慢性萎缩性胃炎。此外，还有一些特殊类型的胃炎，如化学性胃炎、肉芽肿性胃炎、胶原性胃炎、放射性胃炎、感染性胃炎和巨大肥厚性胃炎。	The causes of chronic gastritis include H.pylori infection, autoimmune gastritis, food-induced eosinophilic gastroenteritis, lymphocytic gastritis, and so on. According to the classification system of chronic gastritis, it can be divided into chronic non-atrophic gastritis and chronic atrophic gastritis. In addition, there are some special types of gastritis, such as chemical gastritis, granulomatous gastritis, collagenous gastritis, radiation gastritis, infectious gastritis, and giant hypertrophic gastritis.	慢性胃炎是因胃黏膜长时间炎症刺激引起，主要病因包括幽门螺杆菌感染、长期使用非甾体抗炎药、自身免疫反应、不良饮食、胃酸倒流以及暴露于有害物质。根据病变类型，可分为慢性非萎缩性胃炎和慢性萎缩性胃炎。非萎缩性胃炎表现为胃黏膜炎症，细胞结构相对正常;而萎缩性胃炎伴随不可逆的变化，包括黏膜萎缩和腺体减少。此外，根据病变部位，还可分为全胃炎、胃窦炎、胃体炎等。确切的分类通常需通过胃镜检查和组织活检来确定。及时咨询医生获取确诊和治疗建议是关键。	Chronic gastritis is caused by prolonged inflammatory stimulation of the gastric mucosa. The main causes include *Helicobacter pylori* infection, long-term use of nonsteroidal anti-inflammatory drugs, autoimmune reactions, poor diet, acid reflux, and exposure to harmful substances. According to the type of lesion, it can be divided into chronic non-atrophic gastritis and chronic atrophic gastritis. Non-atrophic gastritis showed inflammation of gastric mucosa with relatively normal cellular structure. In contrast, atrophic gastritis is accompanied by irreversible changes, including mucosal atrophy and gland depletion. In addition, according to the location of the lesion, it can be divided into pangastritis, antral gastritis, gastritis and so on. The exact classification is usually determined by gastroscopy and tissue biopsy. Timely consultation with a doctor for diagnosis and treatment advice is the key.
5.患者应在行根除治疗后复查H.pylori的最佳选择是?	5. What is the best choice for patients to be retested for H.pylori after eradication therapy?	尿素酶呼气试验是患者在行根除治疗后复查H.pylori的最佳选择。	Urease breath test is the best choice for H.pylori reexamination after eradication therapy.	在行幽门螺杆菌(*H. pylori*)根除治疗后，患者复查的最佳选择是通过非侵入性检测方法来确定治疗效果。以下是几种常用的检测方法:尿素呼气试验(UBT): 患者饮用含有标记尿素的液体，如果幽门螺杆菌存在，其代谢会产生标记的二氧化碳，可以通过呼气进行检测。粪便抗原检测: 患者的粪便样本中检测幽门螺杆菌抗原的存在，是一种简便而非侵入性的方法。血清抗体检测: 检测患者血清中的抗体水平，但这种方法不适用于判断治疗效果，因为抗体水平可能在治疗后仍然持续一段时间。胃黏膜活检: 胃镜检查后进行幽门螺杆菌的组织学检测，但这是一种侵入性的方法，一般用于初步诊断。在根除治疗后，建议选择非侵入性的检测方法，其中尿素呼气试验和粪便抗原检测是常用的选择。	After eradication therapy for *Helicobacter pylori* (*H. pylori*), the best option for patients to be followed up is to determine the effect of treatment by non-invasive testing. The following are several commonly used tests: Urea breath test (UBT): The patient drinks a liquid containing labeled urea, and if *H. pylori* is present, its metabolism produces labeled carbon dioxide, which can be detected by exhalation. Stool antigen testing: The patient’s stool sample is tested for the presence of *Helicobacter pylori* antigen, which is a simple and non-invasive method. Serum antibody testing: Tests for antibody levels in a patient’s serum, but this method is not suitable for judging the response to treatment because antibody levels may persist for some time after treatment. Gastric mucosal biopsy: Histological detection of *H. pylori* is performed after gastroscopy, but this is an invasive method and is generally used for initial diagnosis. After eradication therapy, the choice of non-invasive testing methods is recommended, of which urea breath test and stool antigen detection are commonly used options.

### 3.2 Fine-tuned embedding model improved performance

**Figure 4:**
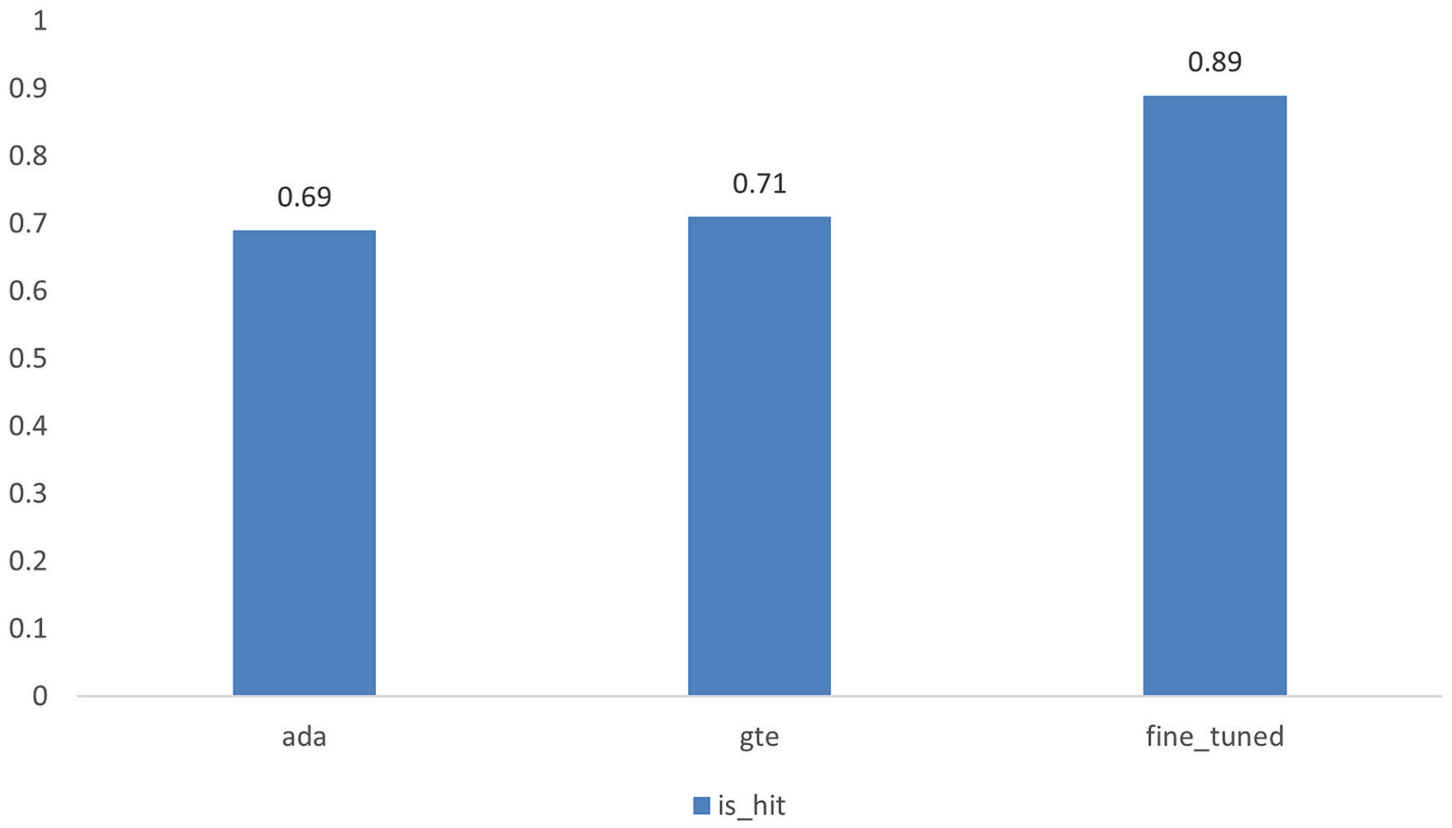
Illustrates the hit rates of the text-embedding-ada-002 model, the gte-base-zh model, and the fine-tuned model.

**Figure 5:**
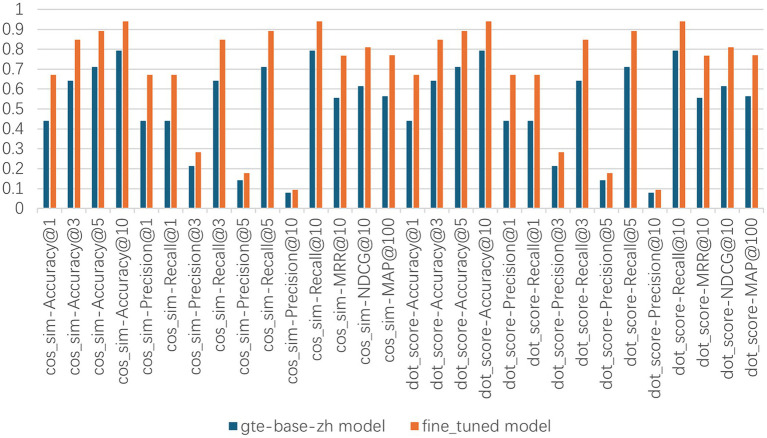
Information retrieval evaluator is a comprehensive metric suite provided by LlamaIndex, showcasing the outcomes of 30 evaluation metrics.

The results of the hit rate are illustrated in Figure 4 and presented in Table 2. Our fine-tuned model exhibits an 18% improvement in performance compared to its base model, gte-base-zh. When contrasted with OpenAI’s embedding model, text-embedding-ada-002, our fine-tuned model demonstrates a 20% enhancement in performance. The results of the Information Retrieval Evaluator evaluation are presented in Figure 5, showing a 21% improvement in performance for the fine-tuned model compared to the base model. Additionally, the fine-tuned model exhibits improvements in each of the 30 evaluation metrics columns.

**Table 2:** The hit rate results for the three embedding models.

Model	is_hit
ada	0.69
gte	0.71
fine_tuned	0.89

### 3.3 RAG evaluation

#### 3.3.1 RAGAs scores

Our Ragas evaluation relies on GPT-3.5-Turbo. To ensure diversity and representativeness in the test set, we meticulously designed the distribution of questions across categories such as “simple,” “inference,” “multi-context,” and “conditional.” Adhering to these guidelines, we curated a test set comprising 20 questions for assessment. The evaluation outcomes of Ragas are consolidated in Table 3. When employing the RAGAS framework to evaluate GastroBot, we attained a context recall rate of 95%, with faithfulness reaching 93.73%. Moreover, the answer relevancy score achieved 92.28%.

**Table 3:** Displays the evaluation results for RAGAS.

RAGAS	Result
Faithfulness	93.73%
context_recall	95%
answer_relevancy	92.28%

#### 3.3.2 SUS scores

To assess the model’s performance, we enlisted 5 professionals with medical expertise and randomly selected 20 questions recommended by GastroBot for evaluation. The experimental results of the SUS scores are detailed in Table 4. In comparison with the other three models, GastroBot scored remarkably high in terms of safety, usability, and smoothness, achieving scores of 2.87, 2.72, and 2.88, respectively. These scores signify that GastroBot’s responses are exceptionally smooth and notably enhance the accessibility of knowledge while maintaining safety.

**Table 4:** Experimental results of SUS score for the models.

	Safety	Usability	Smoothness
ChatGLM-6B	2.58	2.43	2.45
Llama 2	2.13	1.81	2.38
Qwen-7B	2.09	1.78	2.1
**Ours**	**2.87**	**2.72**	**2.88**

Bold values indicate superior performance.

## 4 Discussion

### 4.1 Previous research background

The extensive utilization of LLM in natural language processing showcases remarkable generalization capabilities. However, challenges such as hallucinations and interpretability issues persist in clinical applications. Our research tackles these challenges by introducing the RAG method, which improves the accuracy and relevance of answers by retrieving information from external knowledge sources. RAG has previously proven successful in biomedical fields (16), liver disease research (18), clinical test diagnostics (19), and electrocardiogram data diagnostics (20).

In Wang et al.’s study, the application of the Almanac framework improved the retrieval function of medical guidelines and treatment recommendations, showcasing the potential effectiveness of LLM in clinical decision-making (17). Moreover, Ge et al. (18) utilized RAG technology to develop Li Versa, a specialized model for liver diseases, which offered more targeted answers compared to ChatGPT. These studies robustly support our work, affirming the practical application potential of RAG technology in professional domains. The high prevalence of *Helicobacter pylori* infection in China correlates with the increasing incidence of gastric cancer, ranking third in both incidence and mortality among all cancers (23, 24). Given the substantial patient population and the complexity of managing gastrointestinal diseases, implementing RAG is paramount for enhancing the accuracy of diagnosis and treatment for these diseases.

### 4.2 Novelty discovered

We employed a corpus consisting of 25 guideline documents and 40 relevant literature articles to apply RAG technology in clinical practice within the field of Chinese gastroenterology. By fine-tuning the embedding models, we achieved a significant enhancement in performance. Post fine-tuning, our model exhibited an 18% increase in hit rate compared to the base model gte-base-zh. Furthermore, our fine-tuned model demonstrated a 20% performance improvement compared to OpenAI’s embedding model. Concurrently, leveraging the RAG framework, we developed GastroBot, a Chinese gastroenterology chatbot. Evaluation using the RAGAS framework showcased a context recall rate of 95%, faithfulness of 93.73%, and a high answer relevancy of 92.28%. Human assessments indicated GastroBot’s excellent performance in safety, usability, and smoothness, with scores of 2.87, 2.72, and 2.88, respectively. These findings underscore the significant advantages and innovative potential of RAG technology in addressing clinical challenges.

### 4.3 Explanation of potential drawbacks and limitations

Although RAG technology has shown significant improvements in accuracy and relevance, it still faces inherent limitations. The quality and accuracy of external knowledge sources directly impact the quality of generated responses. In our study, using 25 guideline documents and 40 literature articles, we encountered challenges related to the comprehensiveness and timeliness of knowledge. Responses may lack necessary details or specificity, requiring subsequent clarification. Additionally, responses may sometimes be overly vague or generic, failing to effectively meet user needs. In future work, we intend to explore complex retrieval strategies to address these challenges. It is important to note that our current research primarily focuses on the Chinese region, thus exhibiting geographical limitations. Future research endeavors will strive to enhance the model’s applicability, encompassing broader regions and cultural backgrounds. Furthermore, RAG technology’s reliance on large datasets may hinder its performance in scenarios with limited sample sizes. Subsequent research plans should aim to alleviate these challenges to bolster the model’s resilience and adaptability.

### 4.4 Integration with current problem understanding and advancement

Our research provides a fresh perspective and solution to the information processing challenges faced in today’s clinical environment. Currently, AI chatbots are making significant strides in healthcare, particularly in pain management (37, 38). Inspired by these advancements, we successfully integrated RAG technology, which combines LLM’s reasoning capabilities with domain-specific knowledge retrieval, to develop an AI chatbot tailored to addressing the interpretation and understanding challenges in clinical gastroenterology. This advancement not only deepens our understanding of clinical problem-solving but also holds promise for extending this technology to other clinical domains, especially in underserved regions with limited medical resources, thereby aiding in early diagnosis.

### 4.5 Theoretical hypotheses for future directions and testing

Future research endeavors may concentrate on refining the selection and updating mechanisms of external knowledge sources to ensure the model’s access to the latest and most comprehensive clinical data. To enhance GastroBot’s performance, we intend to explore the utilization of advanced RAG technology. Moreover, we will seek collaboration with experts in gastroenterology to enrich GastroBot’s domain-specific knowledge. Integrating electronic medical record systems into GastroBot is also part of our future enhancement agenda. Additionally, we anticipate the emergence of high-performance LLMs deployable locally, facilitating the deployment of relevant chatbots by researchers in various fields. Fundamentally, our research introduces an innovative solution to the clinical information processing domain and provides valuable insights for future studies. Ongoing improvements and deeper exploration suggest that RAG technology may play a crucial role in the foreseeable future, particularly in fields such as clinical decision support systems.

## Data availability statement

The original data for this research is sourced from the Chinese Medical Journal Full-text Database (https://www.yiigle.com) and China National Knowledge Infrastructure (https://www.cnki.net). Other experimental-related code, exported datasets, and model weights have been released on GitHub (https://github.com/hujili007/ragbot).

## Author contributions

QZ: Writing – original draft. CL: Validation, Writing – review & editing. YD: Data curation, Writing – review & editing. KS: Writing – review & editing. YL: Writing – review & editing. HK: Writing – review & editing. ZG: Writing – review & editing. JS: Writing – review & editing, Investigation. JH: Writing – review & editing.

## Footnotes

^1^ https://www.yiigle.com

^2^ https://www.cnki.net/

^3^ https://www.llamaindex.ai/
